# Micro-Ring Resonator-Based Tunable Vortex Beam Emitter

**DOI:** 10.3390/mi15010034

**Published:** 2023-12-23

**Authors:** Liaisan I. Bakirova, Grigory S. Voronkov, Vladimir S. Lyubopytov, Muhammad A. Butt, Svetlana N. Khonina, Ivan V. Stepanov, Elizaveta P. Grakhova, Ruslan V. Kutluyarov

**Affiliations:** 1 School of Photonics Engineering and Research Advances (SPhERA), Ufa University of Science and Technology, 32, Z. Validi St., 450076 Ufa, Russia; bakirova.li@ugatu.su (L.I.B.); voronkov.gs@ugatu.su (G.S.V.); stepanov.iv@ugatu.su (I.V.S.); grakhova.ep@ugatu.su (E.P.G.);; 2 Samara National Research University, 443086 Samara, Russia; khonina@ipsiras.ru; 3 IPSI-RAS-Branch of the FSRC “Crystallography and Photonics” RAS, 443001 Samara, Russia

**Keywords:** vortex beam, orbital angular momentum, micro-ring resonator, phase change material, critical coupling condition, photonic integrated circuit

## Abstract

Light beams bearing orbital angular momentum (OAM) are used in various scientific and engineering applications, such as microscopy, laser material processing, and optical tweezers. Precise topological charge control is crucial for efficiently using vortex beams in different fields, such as information encoding in optical communications and sensor systems. This work presents a novel method for optimizing an emitting micro-ring resonator (MRR) for emitting vortex beams with variable orders of OAM. The MRR consists of a ring waveguide with periodic structures side-coupled to a bus waveguide. The resonator is tunable due to the phase change material Sb_2_Se_3_ deposited on the ring. This material can change from amorphous to crystalline while changing its refractive index. In the amorphous phase, it is 3.285 + 0*i*, while in the transition to the crystalline phase, it reaches 4.050 + 0*i* at emission wavelength 1550 nm. We used this property to control the vortex beam topological charge. In our study, we optimized the distance between the bus waveguide and the ring waveguide, the bending angle, and the width of the bus waveguide. The optimality criterion was chosen to maximize the flux density of the radiated energy emitted by the resonator. The numerical simulation results proved our method. The proposed approach can be used to optimize optical beam emitters carrying OAM for various applications.

## 1. Introduction

Optical beams bearing orbital angular momentum (OAM) [1], often referred to as vortex beams, represent a vibrant and pivotal domain of investigation, resonating across a spectrum of applications within the realm of information and communication technologies (ICT) [2,3]. The significance of OAM in the realm of optics and photonics is paramount. OAM introduces a unique dimension to the properties of light, enabling it to carry not only energy and momentum but also a quantized amount of angular momentum due to its spatial phase distribution. This remarkable characteristic has far-reaching implications in various fields, such as communication technologies, where OAM-based vortex beams can significantly increase data-carrying capacity and bandwidth in optical fibers [4]. Moreover, OAM’s capacity for fine-grained control and manipulation opens doors to advanced applications in information security, signal processing, and imaging [5,6,7]. Its role extends beyond communication, finding relevance in sensing, imaging, and quantum optics, making OAM a fundamental and versatile tool in the ever-evolving landscape of optical sciences and technologies. Their manifold utility has spurred significant interest and exploration in recent years, with the endeavor to efficiently generate OAM being a focal point of research over the past decade. This research has harnessed a multitude of techniques, including diffractive optics [8], metasurfaces [9], and photonic integrated circuits (PICs) [8].

PICs, in particular, have emerged as a compelling choice for OAM generation, enabling the miniaturization of components, heightened component density, and diminished power consumption [10,11,12]. The imperative of our times underscores the demand for generating vortex beams while simultaneously retaining the ability to dynamically manipulate their topological charge or OAM order [13]. This control opens up a vista of opportunities, especially concerning the secure encoding and decoding of signals in communication lines [14]. Furthermore, the augmentation of bandwidth in fiber-optic communication lines has been substantiated by the incorporation of additional orthogonal OAM bases [15]. Intriguingly, optical vortex beams hold particular promise in the context of sensor applications, offering distinct advantages in various sensing scenarios [16].

The pioneering work of Allen [17] in the field of OAM was followed by a series of research papers that not only confirmed OAM’s generation but also delved into the intricacies of sorting, multiplexing, and demultiplexing these unique optical states. Traditionally, these investigations made use of conventional optical components, such as holograms [18], spatial light modulators (SLMs) [19], and dove prisms [20], among others. Nevertheless, as optical communication systems have evolved towards space-division multiplexing (SDM) and coherence-driven approaches, the integration of OAM manipulation into robust platforms has become increasingly crucial. For instance, Su et al. have demonstrated the feasibility of silicon photonic OAM multiplexing and demultiplexing devices, which employ a free propagation region within a Rowland circle and incorporate waveguide apertures with grating structures [21]. While this approach shows promise, it is important to note that alternative integrated 2D photonic circuits for spatial mode creation have been explored by various other research groups [22,23]. These integrated structures, however, do come with certain limitations, such as relatively higher losses and polarization dependencies, which currently hinder their widespread adoption in transmission systems. In an article [24], Xinlun et al. proposed the use of a micro-ring resonator (MRR) with integrated corner gratings as a vortex beam emitter. It can be noted that this device provides fast switching between the states of OAM by changing the resonant wavelength, and a radiation efficiency of up to 13% was also achieved. However, the tuning range is limited by the range of the tunable laser. Also, the paper emphasizes the necessity of optimizing the coupling field to increase the emission efficiency.

## 2. The Structure of the MRR-Based Vortex Beam Emitter

**Figure 1:**
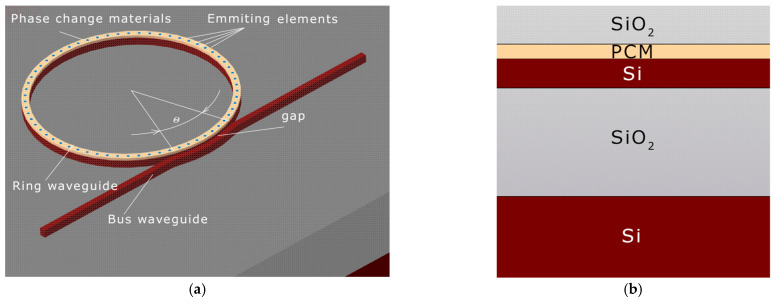
Schematic illustration of the proposed device. (**a**) 3D model: gap—the gap between the ring waveguide and the bus waveguide, θ—bus waveguide angle; (**b**) the layer stack in the model (cross-sectional view).

In this work, we propose an optimization method for an emitting MRR with a periodic grating structure (etched holes) side-coupled to a bus waveguide (single-bus resonator configuration), as shown in Figure 1 [25]. The optimization criterion is the maximization of the energy flux density. The basic principle of vortex beam generation, where the topological charge order *l* satisfies a specific condition, is described in Ref. [26]:

*l = p − gq*, (1)

where *p* is the mode order of the whispering gallery, *q* is the number of the grating elements in the MRR, and *g* is the integer diffraction order, which can be calculated as:

(2) $$\left(n_{eff}-1\right)\frac{2\pi R}{q\lambda }<g<\left(n_{eff}+1\right)\frac{2\pi R}{q\lambda },$$

where *R* is the ring radius, *n_eff_* is the ring waveguide effective index, and *λ* is the operating wavelength. The *p* value is determined as follows [27]:

(3) $$p=\frac{2\pi Rn_{eff}}{\lambda }.$$

Consequently, to change the order of OAM, the effective refractive index of MRR can be changed. Currently, the use of specific coatings on PICs to change the sensor’s functional characteristics is being investigated [28]. We propose to use a phase change material (PCM) as a coating on the ring for the resonator tuning. In this case, the properties of these materials, which allow them to transit between amorphous (disordered) and crystalline (ordered) phases, are utilized. To date, one of the most common compounds used in integrated photonics is Ge_2_Sb_2_Te_5_ (GST) [29]. However, GST is known for its high extinction coefficient value, making it a good choice for switching applications but not optimal for use in radiating structures [30]. Therefore, it is reasonable to use another PCM for such structures. The antimony-based chalcogenide Sb_2_Se_3_ is a material that has ultra-low optical losses at telecommunication wavelengths (around 1550 nm).

Sb_2_Se_3_ also has different complex refractive index values between amorphous (a-PCM) and crystalline (c-PCM) phases. Practical applications of PCM involve the manipulation of parameters such as temperature, electric current, and strain to achieve dynamic control over the refractive index [31]. In our simulations, we used the values of complex refractive indices [32] for different material states: a-PCM = 3.285 + 0*i*, c-PCM = 4.050 + 0*i* at the wavelength *λ* = 1550 nm. We performed all numerical simulations for the silicon-on-insulator (SOI) PIC platform.

According to technological requirements for typical multi-project wafer (MPW) fabrication runs, for the silicon waveguide height of 220 nm, the minimum radius of an MRR on the SOI platform is 5 μm [33] (the width may vary in a wide range, but for wavelengths close to 1550 nm for the single-mode regime, it is close to 400 nm). It is known that to change the topological charge with a change in frequency [34], rings with a radius of more than 25 μm are used, as rings with a smaller radius do not allow for a change in resonances. In our study, we selected a ring radius of 5.5 μm to reach the device’s miniaturization and tested the feasibility of altering the order of the optical vortex beam. The simulation was conducted for MRR with PCM application, as shown in Table 1.

## 3. Optimization Methodology and Simulation

As a standard practice in the MRR design, the initial step involves considering the critical coupling criterion [35]. In a conventional resonator, the ring transmission coefficient should equal the transmission coefficient in the bus waveguide. However, in our case, we have a ring with a low-quality factor, designed for light radiation, so this approach requires adaptation. We propose to divide the overall ring loss *A_ring_* into the ring attenuation loss *A_att_* and the ring radiation loss *A_dots_*. To determine the value of the radiation loss *A_ring_* over the whole ring waveguide, we used the methodology described in Ref. [36]. According to this method, the value numerically obtained for the quartile ring transmission factor *T_qring_* must be raised to the fourth power to find *T_ring_*. This simplifies the numerical modeling and reduces computational resources.

Thus, the critical coupling criterion for the vortex beam emitter is:

(4) $$t=T_{qring}^{4},$$

where *t* is the MRR through port amplitude transmission coefficient.

First, we performed the MRR optimization on the model in the absence of the PCM layer in several steps: optimization of the distance (gap) between the bus waveguide and ring waveguide, optimization of the bus waveguide width to match the phases in the two coupled, and optimization of the bending angle of the bus waveguide. Each optimization stage is presented in detail below.

**Figure 2:**
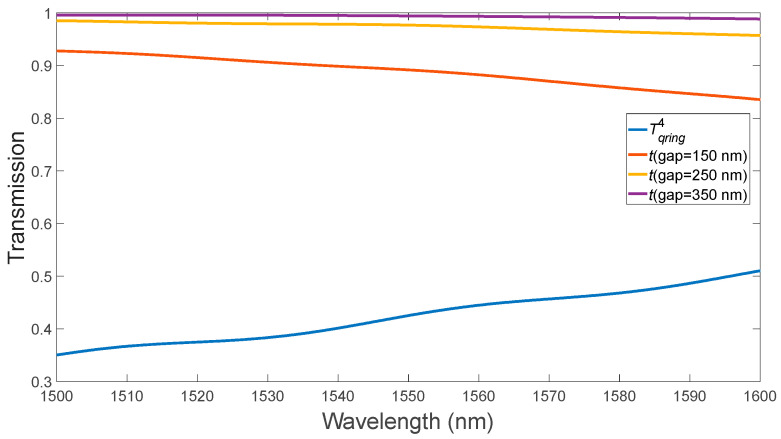
Variation of the MRR gap without PCM layer.

**Figure 3:**
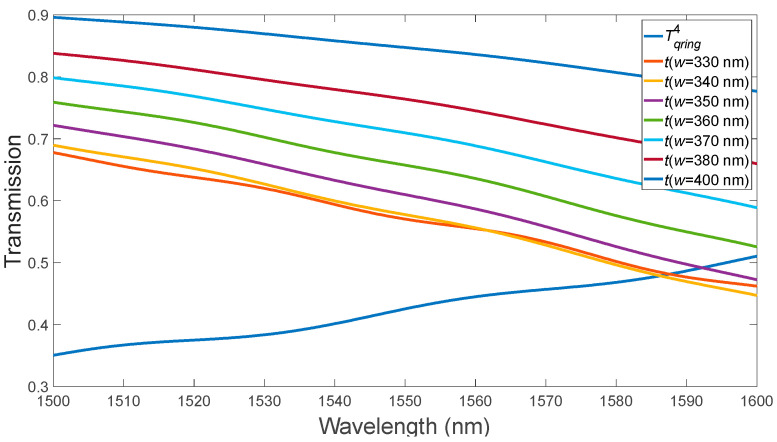
Variation of the bus waveguide width for the MRR in the absence of the PCM layer.

(1) Optimization of the distance between the bus waveguide and the ring (gap) (Figure 2). From Figure 2, it is evident that the optimal gap value was 150 nm. Further reduction of the gap would entail technological difficulties during manufacturing. However, the condition for critical coupling, which ranges from 1500 nm to 1600 nm, is not met.

(2) Optimizing the bus waveguide width to match the phases in the two coupled waveguides (here, the ring waveguide width is constant and equals 400 nm) (Figure 3). We optimized the width of the bus waveguide to match the phases in two coupled waveguides (from the perspective of equal refractive indices of the ring waveguide with etched holes and the bus waveguide, while the width of the ring waveguide remains constant at 400 nm).

**Figure 4:**
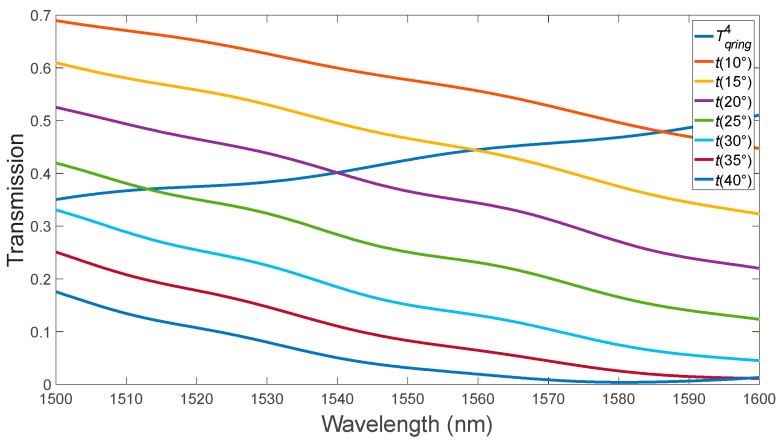
Variation of the bus waveguide bending angle for the MRR in the absence of the PCM layer.

In the second stage, the optimal value of the input waveguide width, 340 nm, was obtained (Figure 3). At a wavelength of 1587 nm, we observe that the critical coupling condition is satisfied. Optimization of the bending angle θ of the bus waveguide in the “pulley-coupling” scheme (Figure 4) [18].

**Figure 5:**
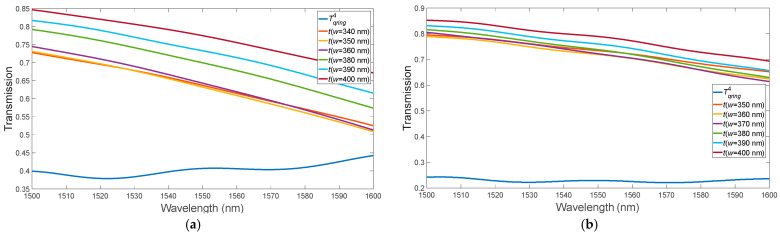
Variation of the MRR with PCM layer: (**a**) a-PCM; (**b**) c-PCM.

**Figure 6:**
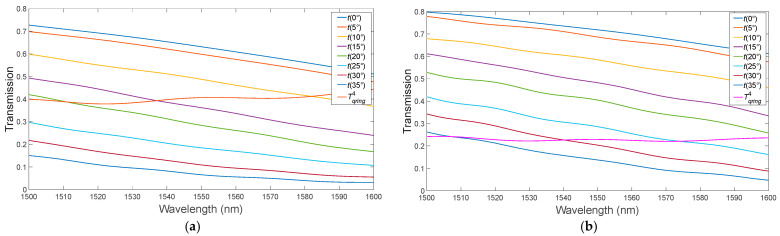
Variation of the bus waveguide bending angle for the MRR with PCM application: (**a**) a-PCM; (**b**) c-PCM.

When we adjusted the bending angle of the bus waveguide, we found that the optimal value was 20°. Under these conditions, the critical connection is achieved at a wavelength of 1540 nm, as illustrated in Figure 4. Next, using the described algorithm, we optimized the MRR with a-PCM and c-PCM coatings (Figure 5 and Figure 6). The optimal gap size, observed in the absence of PCM application, was determined to be 150 nm.

When a material with a phase transition in the a-PCM state is deposited on a ring waveguide, the optimal width of the input waveguide is 350 nm, and in the c-PCM state, it is 370 nm (Figure 5).

The optimal bending angle for the bus waveguide for a-PCM is approximately 15° at a wavelength close to 1550 nm, while for c-PCM it is 30° (Figure 6). This difference is attributed to the distinct refractive indices of the amorphous and crystalline states of PCM. Additionally, the transmission coefficient of the ring waveguide in the a-PCM and c-PCM states differs noticeably due to PCM’s higher refractive index compared to silicon, resulting in the concentration of the field around the PCM layer on the ring’s surface, as depicted in Figure 5 and Figure 6. Table 2 summarizes the MRR parameters satisfying the critical coupling condition, and Finite Difference Time Domain (FDTD) numerical simulations were used to determine the radiation flux power and effective power for various resonant wavelengths in the optimized MRR. It is important to note that the radiative power generally increases at resonances with shorter wavelengths, which can be attributed to the dependence of Rayleigh scattering on wavelength, where the scattering probability is proportional to *λ*^−4^.

**Figure 7:**
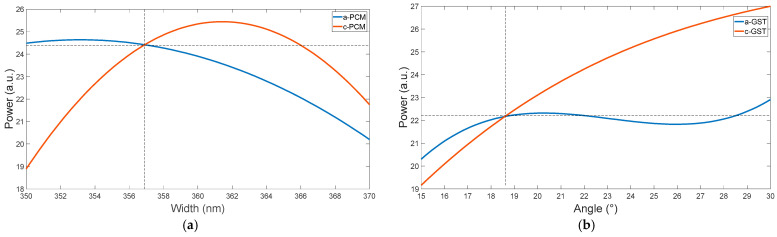
Variation of the radiation power for the MRR at the resonant wavelength of 1545 nm on (**a**) bus waveguide width and (**b**) bus waveguide bending angle.

Following the established algorithm, a multi-factor optimization was performed to attain equivalent emission power for both the a-PCM and c-PCM phases. The results presented in Figure 7 demonstrate that the same power output was achieved at specific values of the bus waveguide width (356.9 nm) and bus waveguide bending angle (18.63°). Based on previous experimental data [37], the standard deviation for typical waveguide width fabrication on the SOI platform can be as high as 3 nm. Therefore, accounting for this error, the optimized model was determined to have a bus waveguide width of 357 nm and a bus waveguide bending angle of 19°. In the amorphous state of a-PCM, the radiation power at the measured parameters was found to be 21.947, while in the crystalline state of c-PCM, it was 23.049. The observed power loss during phase switching was approximately 4.7%, which is considered acceptable.

**Figure 8:**
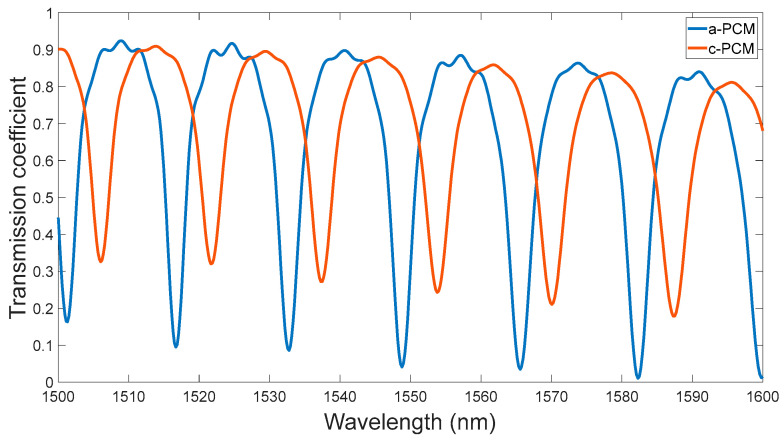
Transmission spectrum of the MRR with application a-PCM and c-PCM (calculated by 3D Lumerical FDTD 2020 R2.4 simulation).

Based on the optimized values, the model characteristics were analyzed, and the MRR transmission spectra for a-PCM and c-PCM are presented in the graphs below (Figure 8). The presented observation indicates that the resonances exhibited by the MRR in the presence of a-PCM and c-PCM possess distinct values. It is noteworthy that the resonances in MRR are periodical, as determined by the FSR (free spectral range) parameter [38]:

(5) $$FSR=\frac{\lambda^{2}}{n_{g}L},$$

**Figure 9:**
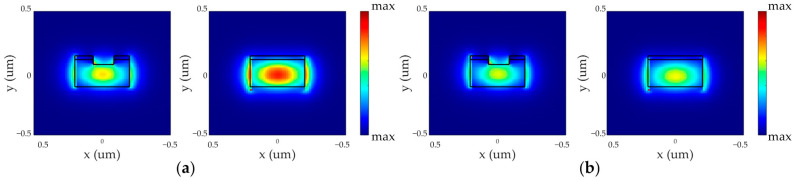
Variation of the radiation power for the MRR at the resonant wavelength of 1545 nm for (**a**) a-PCM and (**b**) c-PCM.

We calculated the FSR using the expression (5) for the MRR with the application of a-PCM = 16.074 nm and c-PCM = 16.03 nm. The simulation results showed that the FSR is a-PCM = 16.77 nm and c-PCM = 16.26 nm. The general picture of the change in the MRR transmission spectrum is shown in Figure 8, and the change in the field pattern due to PCM switching is shown in Figure 9.

## 4. Simulation Results

**Figure 10:**
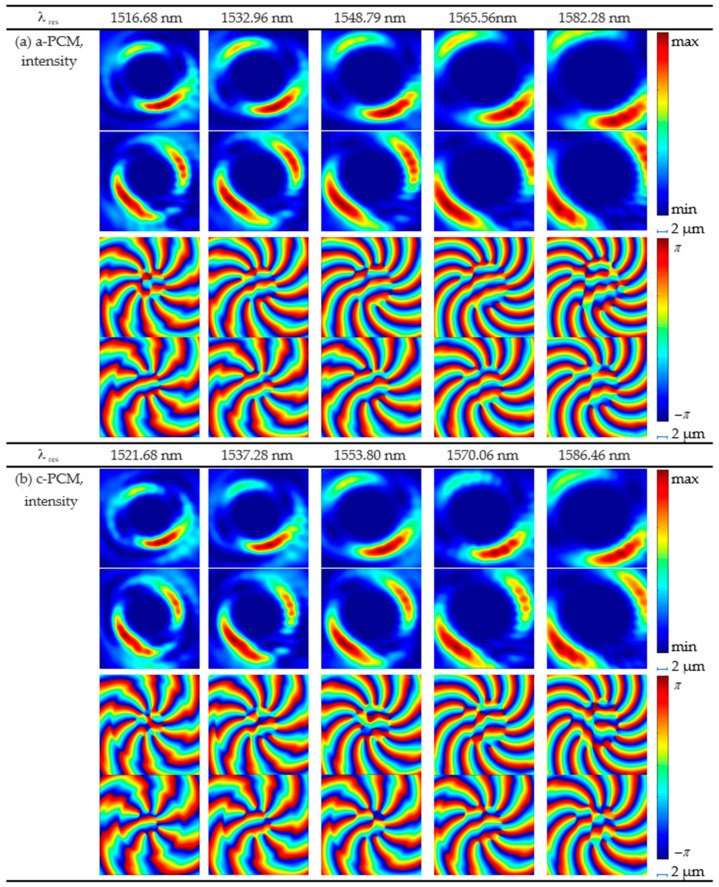
Intensity and phase distributions of the emitted fields from the ring: (**a**) for a-PCM application (**b**) for c-PCM application. (a11–a15) and (a21–a25) are intensities, |*Ex*|^2^ and |*Ey*|^2^, at the given resonant wavelengths, respectively; (a31–a35) and (a41–a45)—phase distribution of the x-component and y-component at the given resonant wavelengths, respectively. Phase distributions are obtained after the field passes through a quarter-wave plate; therefore, the azimuthal order of its x-components is higher, and the azimuthal order of the y-components is lower by one in magnitude than the actual order of the generated vortex beam. Distribution patterns (b11–b15)–(c41–c45) are obtained similarly.

We calculated the topological charge of the OAM after optimization (Figure 10) and compared the results to the simulations carried out using the FDTD method in the Ansys Lumerical 2020 R2.4 environment. The results showed that the OAM order was minus 12 for a-PCM and minus 11 for crystalline. The numerical simulation results showed that at the amorphous phase, the order of OAM was minus 11, and at the complete transition to the crystalline phase, the order of OAM reached minus 10. The analytical solution was close to the numerical simulation results, but further elaboration is required for more accurate calculations.

## 5. Discussion

The proposed MRR tunable vortex emitter utilizing PCM is a significant advancement in the field. Controlling the PCM phase is necessary to change the topological charge. It is important to note that the transition between amorphous and crystalline states only occurs instantly for the entire volume of the material [39]. That means that PCM has transitional forms, making it easy to vary the OAM orders in a wide range of values. An exciting topic for further research is designing a device to influence the material’s phase stage-by-stage with a stage-by-stage transition.

**Figure 11:**
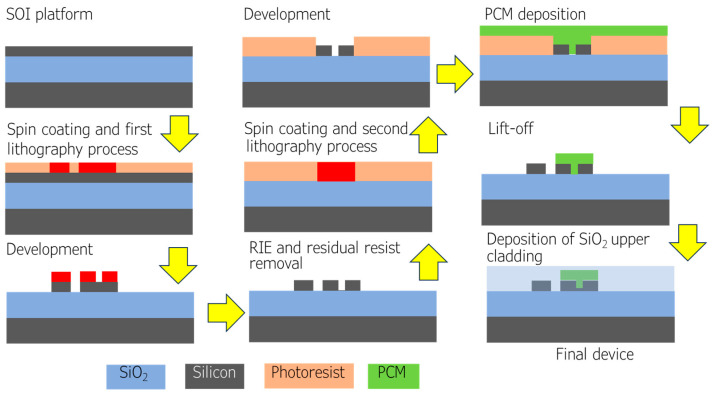
The cross-sectional view of the suggested fabrication process.

The proposed device structure is easy to fabricate in a two-step fabrication process. The first step is preparing the structure of the SOI substrate platform, which consists of a 2 μm-thick silicon substrate (Si) topped with a 3 μm-thick SiO_2_ insulator and an active top layer of silicon measuring 220 nm in thickness. After that, one should use the electron beam lithography process to create the necessary lead-in and ring waveguide models with etched holes by employing reactive ion etching (RIE). Then, in the second lithography process, a positive photoresist should be applied to the entire wafer surface, matching the height of the ring and bus waveguide, and PCM should be deposited using a magnetron sputter. One can use the manufacturing process described in Ref. [40] to deposit PCM on the SOI platform. Finally, the desired structure could be achieved after removing the photoresist via the lift-off process and depositing an upper cladding SiO_2_ layer on top of the device. Figure 11 illustrates the detailed manufacturing process. In practical applications, a 10 nm thick layer of indium tin oxide (ITO) should be applied after PCM deposition; this will help prevent oxidation of the PCM layer [41].

It should be mentioned that the proposed device tunes OAM orders only by one. However, one can easily improve this parameter by simply increasing the radius of the MRR emitter based on Equations (1)–(3) and applying the optimization algorithm described in Section 3.

**Figure 12:**
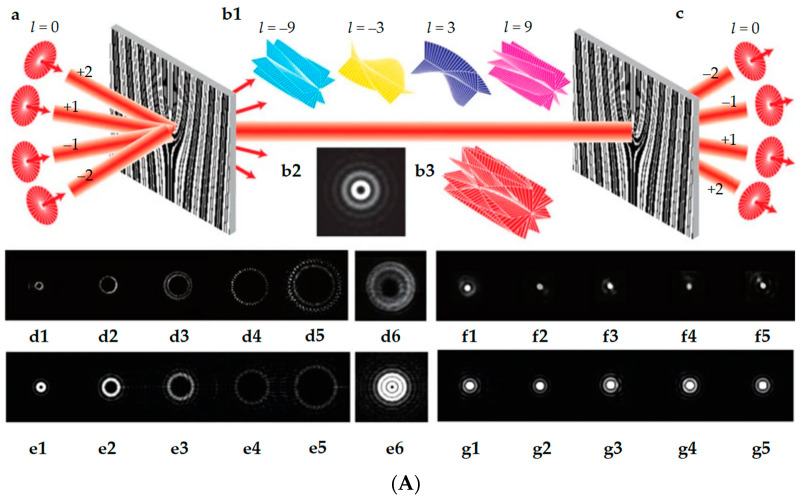

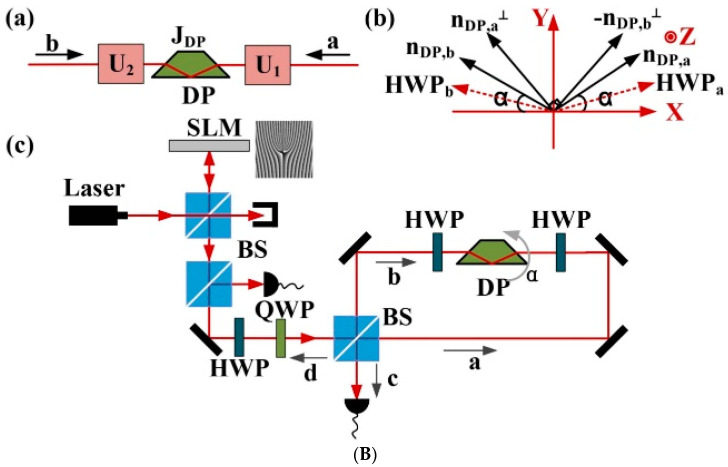
Principle of operation of a circuit for controlling the order of OAM using Dammann lattice (**A**): (**a**) Gaussian beams incident on the grating at its diffraction angles. (**b**) The combined coaxial OV beam with multiple OAM states (**b1**) propagates in free space. (**b2**,**b3**) The simulated intensity pattern and wavefront of the OV beam, respectively. (**c**) The OAM channels are converted into Gaussian beams and are separated spatially for detection. (**d1**–**d5**) Measured intensity profiles of the OAM beams with topological charges (*l* =3, −9, 15, −21, 27). (**d6**) Measured coaxial OV beam with 10 OAM states (*l* =±3, ±9, ±15, ±21, ±27). (**e**) The corresponding modeling results for d. (**f**) The measured Gaussian beams after DEMUX of the OAM channels in d. (**g**) The corresponding modeling results for f. DOVG, Dammann optical vortex grating; MUX/DEMUX, multiplexing/demultiplexing; OV, optical vortex [45], and Dove prism (**B**): (**a**) The unitary transformations to rotate the polarization. (**b**) *n*_*DP*,*a*(*b*)_ and *n*^⊥^_*DP*,*a*(*b*)_ consist the coordinate system of the DP for direction a (**b**). *HWP*^*a*(*b*)^_*i*_ is the fast axis of the HWP in the BS SPSI for direction a (**b**). (**c**) The proof-of-principle experimental setup of the modified BS SPSI. SLM: spatial light modulator; QWP: quarter-wave plate [46].

Controlling the OAM order of an optical vortex beam can be applied to various ICT applications. In recent years, OAM has been an important resource in increasing the throughput of information channels in both classical and quantum information processing [42,43,44]. Among the existing solutions, the advantages of our proposed setup are the compactness of the setup, fast tuning of the OAM order, and the possibility of obtaining high orders. For example, in Ref. [45], the authors demonstrate the generation, transmission, and simultaneous detection of the OAM order by using Dammann optical vortex gratings (Figure 1a). However, such a setup has a limited detection range due to low diffraction efficiency. In Ref. [46], the authors used a Dove prism (Figure 12B) to control the order of the OAM. The bulky optical elements in this design can lead to practical problems in terms of portability and integration into compact systems.

Another option for the OAM generation is presented by a tunable microlaser [47]. The authors used two pump lasers to control beam phase distribution in this work. The main advantages of the proposed design are the small switching time (picosecond scale) and a wide range of OAM order tuning. However, the proposed scheme requires two synchronized nanosecond lasers, which increases manufacturing and controlling complexity.

VCSELs (vertical cavity surface emitting laser) also have great potential in OAM generation [48,49]. In Ref. [50], the authors described how to change the OAM order using lead halide perovskite vortex microlasers. However, the perovskite emission wavelength is limited from 400 nm to 1000 nm [51,52], so these materials are unsuitable for the traditional telecommunication wavelengths close to 1550 nm. The same problem has OAM generation based on 2D van der Waals materials [53], whose operation wavelengths are no more than 800 nm [54].

Also, it is possible to apply metasurfaces for OAM generation [55,56]. These solutions provide an extremely wide operating range of over 100 nm [55]. However, the authors suggest only geometry changes to provide OAM order tuning, which can only be realized with microelectromechanical systems with relatively high switching time (about 1–2 μs) and control voltage (about 30–40 V) [57].

## 6. Conclusions

In pursuit of optimizing the performance of MRR in generating vortex beams, we have introduced a novel methodology that leverages etched gratings. This innovative approach helps to achieve maximal radiation power output, aligning with the critical coupling condition. In our study, the radiation power of our model was initially 25 a.u. before optimization with a-PCM. Upon switching to c-PCM, the radiation power decreased to 15 a.u. However, implementing a new optimization method achieved a radiation power of 31 a.u. for both PCM phases.

Furthermore, a previous work [25] measured the radiation power at 9.8 a.u when the authors integrated a p-n diode into the MRR to control the vortex beam topological charge. Our research included simulations of an MRR-based vortex beam emitter without the coating, combining it with a phase change material. PCM offers swift and precise control over the optical properties, facilitating manipulation of the emitted beam’s orbital angular momentum order. The switching time between phase transitions PCM reaches up to 500 ns [58], while when using the thermo–optical effect, the switching time is 20 μs [43]. As a result, we advocate for the continued development of MRR technology incorporating PCM in information and communication technology (ICT) systems. Moreover, the promising application of this approach extends to sensing systems, where it can concurrently measure the refractive index of the environment and the OAM order of the emitted beams, presenting a versatile solution for a wide array of optical sensing applications.

## Figures and Tables

**Table 1:** MRR parameters.

Name	Value
Ring radius	5.5 μm
Number of holes	63
Hole depth	70 nm
Hole diameter	150 nm
Ring waveguide width	400 nm
Ring waveguide thickness (Si)	220 nm
Application thickness of PCM	30 nm
SiO_2_ thickness	5 μm
Substrate thickness (Si)	2 μm

**Table 2:** MRR optimized parameters.

	Gap (nm)	Angle (°)	Width (nm)	Holes Depth (nm)	Wavelength (nm)	Radiation Output Power (a.u.)	$\mathrm{Effective}\;\mathrm{Power},\;\times 10^{-3}$ (a.u./m^2^)
Without	100	20	340	70	1516.11	30.472	0.116
PCM					1529.64	27.174	0.104
					1545.99	29.484	0.130
					1562.70	21.941	0.083
					1579.99	24.671	0.094
a-PCM	150	15	350	70	1517.07	22.23	0.101
					1532.96	20.67	0.079
					1549.19	24.48	0.093
					1565.97	17.62	0.067
					1582.97	26.24	0.098
c-PCM	150	30	370	70	1521.49	30.07	0.115
					1537.08	24.72	0.945
					1553.40	28.66	0.110
					1569.65	21.75	0.083
					1587.09	26.36	0.101

## Data Availability

Data are contained within the article.
